# 

**Published:** 1894-06

**Authors:** 


					﻿Vol XIV.
JUNE, 1894.
No. 6.
THE
pDMEEOPATHlC pHY^lClAJI,
A Monthly Journal of Homoeopathic Materia Medica
and Clinical Medicine. _
“ He it a freeman whom truth makes	d all arbeside."
“Seek the Truth: come whence it may, costjpfjjtt util."
D PVBL^HEb BY
Walt™ m.	d.
PHILADELPHIA :
ISTo. 1231 LOCUST STREET.
LONDON:
ALFRED HEATH & CO., Bole Agents fob Great Britain,
114 Ebury Street, Eaton Square.
Yearly Subscription, $2.50, invariably in advance. Single numbers, 25 ets.
Entered at Ute Philadelphia Peat-OOiee aa Seeond Claaa Mail Matter.
				

